# Influence of Lactose Supplementation on Regulation of *Streptococcus thermophilus* on Gut Microbiota

**DOI:** 10.3390/nu15224767

**Published:** 2023-11-13

**Authors:** Peng Yu, Yuqi Pan, Zhiwen Pei, Min Guo, Bo Yang, Yuan-Kun Lee, Xiaoming Liu, Jianxin Zhao, Hao Zhang, Wei Chen

**Affiliations:** 1 State Key Laboratory of Food Science and Technology, Jiangnan University, Wuxi 214122, China6210113068@stu.jiangnan.edu.cn (Z.P.); bo.yang@jiangnan.edu.cn (B.Y.); zhaojianxin@jiangnan.edu.cn (J.Z.); zhanghao61@jiangnan.edu.cn (H.Z.); chenwei66@jiangnan.edu.cn (W.C.); 2 School of Food Science and Technology, Jiangnan University, Wuxi 214122, China; guomin@jiangnan.edu.cn; 3 International Joint Research Laboratory for Pharmabiotics & Antibiotic Resistance, Jiangnan University, Wuxi 214122, China; micleeyk@nus.edu.sg; 4 Department of Microbiology and Immunology, Yong Loo Lin School of Medicine, National University of Singapore, Singapore 117597, Singapore; 5 Wuxi Translational Medicine Research Center, Wuxi 214122, China; 6 Jiangsu Translational Medicine Research Institute, Wuxi Branch, Wuxi 214122, China; 7 National Engineering Research Centre for Functional Food, Jiangnan University, Wuxi 214122, China

**Keywords:** *S. thermophilus*, lactose, gut microbiota, metabolome

## Abstract

It has been found that *Streptococcus thermophilus* (*S. thermophilus*) influenced the gut microbiota and host metabolism with strain specificity in C57BL/6J mice in the previous study, though it remains unclear whether lactose as a dietary factor associated with dairy consumption is involved as the mediator in the interaction. In the present study, integrated analysis of 16S rRNA gene sequencing and untargeted metabolomics by liquid chromatography–mass spectrometry of fecal samples in C57BL/6J mice was applied to evaluate the effect of lactose on the regulation of gut microbiota by two *S. thermophilus* strains (4M6 and DYNDL13-4). The results showed that the influence of lactose supplementation on gut microbiota induced by *S. thermophilus* ingestion was strain-specific. Although two *S. thermophilus* strains ingestion introduced similar perturbations in the fecal microbiota and gut microbial metabolism, the regulation of DYNDL13-4 on the gut microbiota and metabolism was more affected by lactose than 4M6. More specifically, lactose and 4M6 supplementation mainly enriched pathways of d-glutamine and d-glutamate metabolism, alanine, aspartate, and glutamate metabolism, and tryptophan and phenylalanine metabolism in the gut, whereas 4M6 only enriched tryptophan and phenylalanine metabolism. DYNDL13-4-L (DYNDL13-4 with lactose) had significant effects on sulfur, taurine, and hypotaurine metabolism in the gut and on phenylalanine, tyrosine, tryptophan biosynthesis, and linoleic acid metabolism in serum relative to the DYNDL13-4. Our study demonstrated the strain-specific effect of lactose and *S. thermophilus* supplementation on gut microbiota and host metabolism. However, considering the complexity of the gut microbiota, further research is necessary to provide insights to facilitate the design of personalized fermented milk products as a dietary therapeutic strategy for improving host health.

## 1. Introduction

*Streptococcus thermophilus* (*S. thermophilus*) is a major dairy starter with rapid acidification capability used for the manufacture of yogurt and cheese. Previous studies have reported the beneficial impacts of *S. thermophilus* strains on the host health, such as improving lactose digestion, alleviating intestinal mucositis, and preventing colorectal tumorigenesis [1,2], demonstrating alteration of gut microbiota after the intervention of the *S. thermophilus* strains. In addition, probiotic combinations that included *S. thermophilus* strains have been found to have a regulatory effect on the host. The combination of *S. thermophilus* and *Bifidobacterium lactis* given to healthy infants aged 3 to 24 months lowered the risk of their colitis [3]. The probiotic bacterial mixture, including *S. thermophilus,* significantly reduced the diarrhea time by 45 h than the oral rehydration solution alone [4]. Clinical research indicated that probiotic mixes contained *Bifidobacterium animalis* subsp. *lactis*, *Streptococcus thermophilus*, *Lactobacillus bulgaricus*, and *Lactococcus lactis* subsp *Lactis* could regulate the responsiveness of an extensive brain network in healthy women [5].

Lactose is an exclusive constituent of mammalian milk, which has a concentration of approximately 4.6 g/100 mL in bovine milk [6]. It has been observed that lactose does not undergo entire metabolism and absorption in the small intestine, and a proportion of dietary lactose might reach the colon [7]. Dietary lactose supplementation could improve gut health and enhance host immunity. Recent studies have indicated that lactose impacts the composition and metabolome of the gut microbiota [8,9,10]. Previous human studies have discovered a positive connection between lactose consumption and the presence of *Bifidobacterium* in adult fecal samples [11]. Lactose treatment was observed to increase the abundance of *Bifidobacterium* and *Lactobacillus*, decrease the abundance of pathogens, and raise levels of acetate and lactate in the infant’s gut in vitro [12,13]. Furthermore, lactose in human milk stimulates the transcription of cathelicidin antimicrobial peptides and then exhibits immunomodulatory properties in the neonate [9]. 

*S. thermophilus* prefers lactose to other sugars as its primary carbon and energy source. A previous study has shown that lactose consumption contributed to the rapid and high level of *S. thermophilus* LMD9 colonization in the fecal samples of gnotobiotic rats [14]. Our previous research demonstrated that lactose could protect the bile salt stress response of *S. thermophilus* in a strain-independent manner in vitro [15]. Furthermore, we have investigated the effect on the gut microbiota and host metabolism in C57BL/6J mice by multiple *S. thermophilus* strains based on inter-strain differences. Previously, it has been demonstrated that consumption of *S. thermophilus* DYNDL13-4 and DQHXNQ38M61 led to greater alterations in amino acid and lipid metabolism when compared to LMD9 and 4M6. This could be linked to the increased presence of *Bifidobacterium*, *Coriobacteriaceae UCG-002*, *Rikenellaceae RC9 gut group*, and *Lactobacillus* in the DYNDL13-4 and DQHXNQ38M61 group. Meanwhile, DYNDL13-4 and DQHXNQ38M61 are phylogenetically distinct from 4M6 and LMD9 based on genomic analysis. However, it remains unclear if lactose supplementation can influence the gut microbiota and host metabolism driven by *S. thermophilus* in vivo. In this research, the effect of lactose on the regulation of gut microbiota and metabolism in C57BL/6J mice by two *S. thermophilus* strains was evaluated. 

## 2. Materials and Methods

### 2.1. Mice 

Malespecific pathogen-free (SPF) C57BL/6J mice (age 7–8 weeks) were obtained from the Beijing Vital River Laboratory Animal Technology Co., Ltd. (Beijing, China). All the male C57BL/6J mice were housed in the SPF environment in the animal experimental center of the Department of Food Science and Technology, Jiangnan University, Wuxi, China. The mice were kept at 22 °C and were subjected to a standard 12 h/12 h light/dark cycle. Before experiments, they were fed adaptively for a week. All animals had free access to chow diet and water. The experimental procedures were conducted strictly by the guidelines of the Ethics Committee of Jiangnan University, China (JN. No20220915c1121202[333]).

### 2.2. Experimental Design 

The mice were randomly assigned to six treatment groups (*n* = 12 per group) as follows: the control was orally gavaged with 0.9% saline, the two *S. thermophilus* strains groups were orally gavaged with a dose of 10^9^ CFU/d for each mouse (referred to as 4M6 and DYNDL13-4), the lactose group was orally gavaged with a dose of 4 g/kg BW/d (referred to as lactose), and the combination groups were orally gavaged with 4 g/kg BW/d lactose and *S. thermophilus* strain (referred to as 4M6-L and DYNDL13-4-L). For *S. thermophilus* preparation, the strains were cultured in M17 broth for 12 h, respectively, and then resuspended in sterile saline to a concentration of 5 × 10^9^ CFU/mL. For lactose preparation, lactose was dissolved into the sterile saline to a concentration of 40% (*w*/*w*). All mice were subjected to the interventions for 28 days. At the end of the experiment, we placed each mouse individually in the sterilized and clean cage and then collected fresh feces using Eppendorf tubes. In addition, we euthanized the mice in each group on day 28 and collected the blood samples. The fecal and serum samples were frozen at −80 °C for DNA extraction and metabolome analysis.

### 2.3. 16S rRNA Gene Sequencing

Fecal DNA was extracted using a FastDNA Spin Kit (MP Biomedicals, Solon, OH, USA). Using primers as previously described [16], the V3-V4 region of the 16S ribosomal RNA gene was amplified. Alpha and beta diversity were determined via the MicrobiomeAnalyst platform [17,18]. The difference in the abundance of bacterial taxa among groups was determined through linear discriminant analysis (LDA).

### 2.4. Metabolite Analysis of Fecal and Serum Samples

Fecal and serum extracts were prepared for metabolite profiling as previously described [19]. In brief, 100 mg thawed fecal samples were placed in an Eppendorf tube with the sterilized beads added and extracted with 800 μL ice-cold methanol. The samples were homogenized and centrifuged at 12,000× *g* for 15 min. A 400 μL portion of the supernatant was transferred and subjected to drying using a vacuum evaporator. For serum, a volume of 100 μL was aliquoted from each sample into the Eppendorf tube. Subsequently, 400 μL of ice-cold methanol was added to the aliquoted serum, followed by vortexing and incubation at −20 °C for 1 h. The supernatant of extracts was obtained by centrifugation and then dried as described above. The dried extracts from feces and serum were resuspended in 200 and 100 μL methanol/water (4:1, *v*/*v*) for injection, respectively. To prepare the quality control (QC) sample, aliquots of the feces and serum samples were pooled, respectively.

Extracts were separated by UHPLC and analyzed on a high-resolution Q-Exactive mass spectrometer. Electrospray ionization (ESI) was used to detect positive ions and negative ions. In the sample queue, a QC sample was set up in 10 experimental samples at intervals to correct the repeatability of the analysis. The raw data were uploaded to Compound Discovery 3.3 (Thermo Fisher Scientific, Waltham, MA, USA) with a metabolomics analysis pipeline, and tables with retention time, *m/z*, and peak area were generated for further analysis.

### 2.5. Statistical Analyses

Statistical analyses were performed using the GraphPad Prism 8.0. The one-way ANOVA or two-way ANOVA was used for statistical analysis. Tukey’s test was applied, and a *p*-value less than 0.05 indicated statistical significance.

## 3. Results

### 3.1. Effect of Lactose Supplementation on Gut Microbiota Induced by S. thermophilus Ingestion Is Strain-Specific

**Figure 1:**
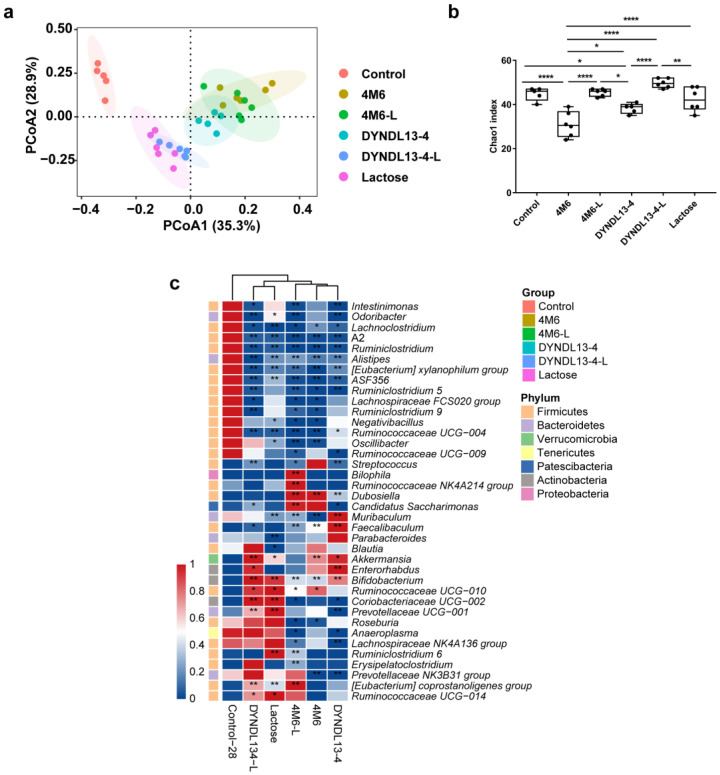
Effect of lactose supplementation on gut microbiota induced by *S. thermophilus* ingestion is strain-specific. (**a**) PCoA plot based on Bray–Curtis distance of taxonomic profiles of fecal samples on day 28; (**b**) Chao index of α-diversity of the fecal microbiota after 28 days of intervention. One-way ANOVA with Tukey’s multiple comparisons; * *p* < 0.05, ** *p* < 0.01, **** *p* < 0.0001; (**c**) The heatmap of genus-level taxa with LEfSe analysis on day 28.

The changes in intestinal microbiota were further explored from fecal samples collected from the mice administered with *S. thermophilus* strains with and without lactose supplementation (4M6, 4M6-L, DYNDL13-4, and DYNDL13-4-L). PCoA of Bray‒Curtis distance of genus-level taxonomic profiles of these samples showed that all interventions altered the structure of the indigenous gut microbiota after 28 days of treatments (Figure 1a). Among these five groups, samples in the 4M6, DYNDL13-4, and 4M6-L groups showed a clear clustering, whereas the DYNDL13-4-L and lactose groups clustered together. This indicated that the ingestion of two *S. thermophilus* strains (4M6 and DYNDL13-4) introduced similar perturbations in the indigenous microbial community structure, and the regulation of DYNDL13-4 on gut microbiota was more affected by lactose than 4M6. Additionally, both 4M6 and DYNDL13-4 decreased alpha diversity relative to the control group, and those were restored under the supplement of lactose (Figure 1b).

The abundance of seven genera, including *Lachnoclostridium*, *A2*, *Ruminiclostridium*, *Alistipes*, *[Eubacterium] xylanophilum group*, *ASF356*, and *Ruminococcaceae UCG-004* decreased compared to the control, with the abundance of only one common genera increased in all the treatments, i.e., *Bifidobacterium* (Figure 1c). 4M6 and DYNDL13-4 shared common modulation effects on the gut microbiota of mice, such as increased abundance of *Bifidobacterium*, *Akkermansia*, *Dubosiella*, and *Faecalibaculum*, and decreased *Prevotellaceae NK3B31 group* and *Ruminiclostridium 5*, with variation in 16 genera between the treatments. With the supplementation of lactose and 4M6, the abundance of *Bilophila*, *Ruminococcaceae NK4A214 group*, *Candidatus Saccharimonas*, and *[Eubacterium] coprostanoligenes group* further increased in 4M6-L. On the other hand, the supplementation of lactose with DYNDL13-4 resulted in a similar pattern of gut microbiota as the lactose group, with increased abundance of ten genera in DYNDL13-4-L (e.g., *Ruminococcaceae UCG-010*, *Coriobacteriaceae UCG-002*, *Prevotellaceae UCG-001*, and *[Eubacterium] coprostanoligenes group*). Additionally, nine genera in 4M6-L and two genera in DYNDL13-4-L that had no significance in corresponding *S. thermophilus* strains and lactose group were affected notably compared to the control. This may be a complex regulation of gut microbiota by the combination of lactose and strains.

### 3.2. Effect of Lactose Supplementation on Fecal Metabolic Profile Induced by S. thermophilus Ingestion Is Similar to Gut Microbiota

**Figure 2:**
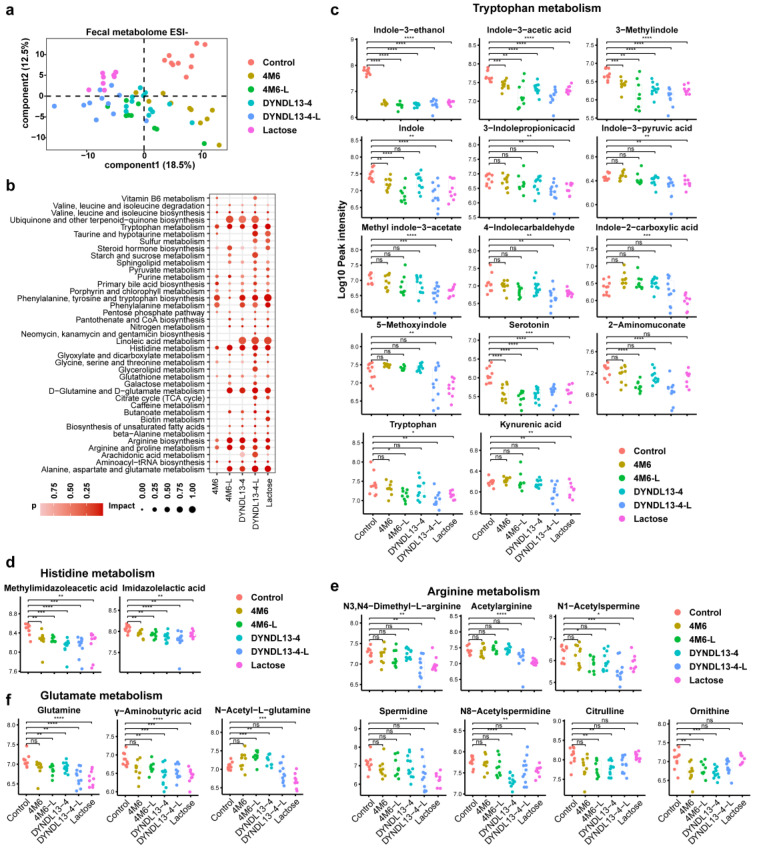
Effect of lactose supplementation on fecal metabolic profile induced by *S. thermophilus* ingestion. (**a**) PLS-DA score plots based on the fecal metabolomic profiles in negative ionization modes; (**b**) the enrichment pathways established using 84 metabolites in fecal samples on day 28 between each treatment and the control group; (**c**–**f**) Box and Whisker plots of fecal metabolites on tryptophan (**c**), histidine (**d**), arginine (**e**), and glutamate metabolism (**f**). * *p* < 0.05, ** *p* < 0.01, *** *p* < 0.001, **** *p* < 0.0001.3.3. Effect of Lactose Supplementation on Serum Metabolome Induced by *S. thermophilus* Ingestion Is Strain-Specific.

To characterize the effect of *S. thermophilus* strains with and without lactose supplementation on metabolic profile in gut microbiota, PLS-DA was performed on the fecal metabolome data (Figure 2a and Figure S1a). The results revealed that all the treatments altered the fecal metabolic profile compared to the control. The differential metabolites between each treatment and the control group are shown in Figure S1c. In line with fecal microbiota, *S. thermophilus* strain groups (4M6 and DYNDL13-4), as well as 4M6-L, clustered closely, whereas DYNDL13-4-L and lactose group clustered more closely (Figure 2a and Figure S1c). There were 14 commonly changed compounds among the five treatment groups (Figure S2a). Lactose intervention resulted in the most abundant alterations (64 specific metabolites) in feces, with the least metabolite changes in the two *S. thermophilus* strain groups (10 specific metabolites by 4M6 and 19 by DYNDL13-4). Lactose supplementation with *S. thermophilus* strains increased the number of altered metabolites in the feces of mice.

Among the differentially abundant pathways, phenylalanine, tyrosine and tryptophan biosynthesis and metabolism, arginine biosynthesis and metabolism, and histidine metabolism were commonly enriched pathways in fecal samples of the five intervention groups (Figure 2b). Lactose supplementation enriched more pathways during the ingestion of *S. thermophilus* strains, with differentiated responses on 4M6 and DYNDL13-4, respectively. 4M6-L significantly enriched pathways of d-glutamine and d-glutamate metabolism, alanine, aspartate, and glutamate metabolism, and DYNDL13-4-L had significant effects on sulfur, taurine, and hypotaurine metabolism when compared to their corresponding strain groups (Figure 2b).

Among the metabolites of tryptophan metabolism in feces, indole-3-ethanol, indole-3-acetic acid, 3-methylindole, and serotonin were reduced in five treatment groups (Figure 2c). 2-Aminomuconate, involved in kynurenine catabolic metabolism, was lowered only in 4M6-L and DYNDL13-4-L, with a reduction of L-tryptophan in groups containing lactose consumption (lactose, 4M6-L, and DYNDL13-4-L). Moreover, indole derivatives (including indole, 3-indole propionic acid, indole-3-pyruvic acid, methyl indole-3-acetate, and 4-indolecarbaldehyde) and kynurenic acid decreased in DYNDL13-4-L, which had a similar trend with those in lactose group. In addition, other indole derivatives, such as indole-2-carboxylic acid and 5-methoxyindole, decreased exclusively in the lactose group. These results revealed that tryptophan metabolism was down-regulated to varying degrees by five treatment groups, and lactose supplementation enhanced these changes, especially in indole derivatives, with DYNDL13-4 more pronounced than 4M6. What is more, metabolites involved in arginine, histidine, and glutamate metabolism in DYNDL13-4 were also more influenced by lactose supplementation than those in 4M6 in feces (Figure 2d–f).

**Figure 3:**
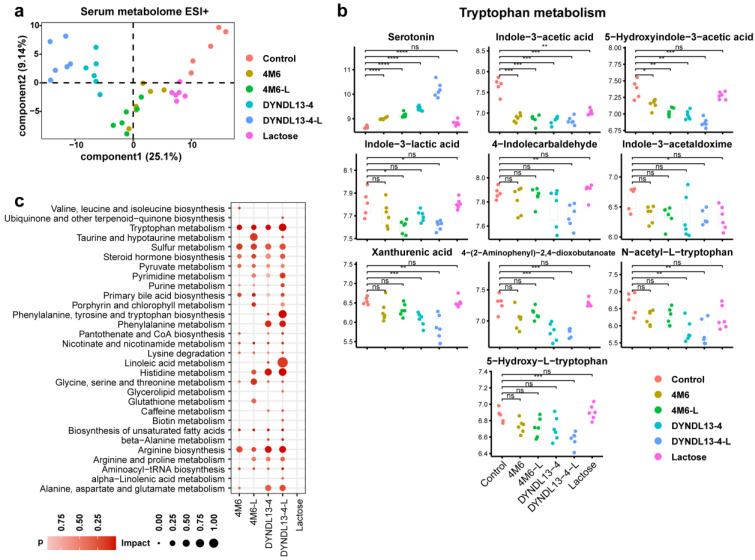
Effect of lactose supplementation on serum metabolome induced by *S. thermophilus* ingestion is strain-specific. (**a**) PLS-DA score plots based on the serum metabolomic profiles in positive ionization modes; (**b**) Box and Whisker plots of serum metabolites on tryptophan metabolism; (**c**) the enrichment pathways established using 84 metabolites in serum samples on day 28 between each treatment and the control group. * *p* < 0.05, ** *p* < 0.01, *** *p* < 0.001, **** *p* < 0.0001. Pathway enrichment analysis revealed that tryptophan, sulfur, pyruvate metabolism, steroid hormone, unsaturated fatty acids, and arginine biosynthesis were enriched in the serum of the strain treatment groups (4M6, DYNDL13-4, 4M6-L, and DYNDL13-4-L) compared to the control group. More pathways were enriched in *S. thermophilus* strain groups with lactose supplement than the corresponding strain groups. The enrichment of taurine and porphyrin metabolism was observed in 4M6-L and DYNDL13-4-L, with no change in 4M6 and DYNDL13-4. Additionally, 4M6-L significantly enriched histidine and arginine metabolism relative to the 4M6, and DYNDL13-4-L remarkably enriched phenylalanine, tyrosine, and tryptophan biosynthesis and linoleic acid metabolism relative to the DYNDL13-4 (Figure 3c).

A total of 525 metabolites were detected in serum, of which 229 were identified in the negative mode and 296 in the positive mode. In contrast to fecal data, samples in the lactose group could not be discriminated from that in the control in serum metabolome according to PLS-DA plots, *S. thermophilus* strain samples with or without lactose supplement, especially DYNDL13-4 and DYNDL13-4-L, were separated from the control (Figure 3a and Figure S1b). In the case of lactose supplementation, serum metabolic pattern was more influenced in DYNDL13-4 than 4M6 (Figure 3a and Figure S1b,d). Two metabolites (including N-isovalerylglycine and indole-3-acetic acid) were shared by DYNDL13-4, 4M6, DYNDL13-4-L, 4M6-L, and the lactose group. Among these five groups, DYNDL13-4 (8 specific metabolites) and DYNDL13-4-L (38 specific metabolites altered more metabolites than 4M6 (4 specific metabolites) and 4M6-L (7 specific metabolites), with no specific alteration observed as a result of lactose consumption. Like fecal data, the lactose supplement promoted more changes in serum metabolome during *S. thermophilus* strains consumption.

In view of metabolites on tryptophan metabolism in serum among four groups (4M6, DYNDL13-4, 4M6-L, and DYNDL13-4-L), serotonin was elevated, whereas indole-3-acetic acid and 5-hydroxyindole-3-acetic acid were generally reduced compared to the control. Indole-3-lactic acid decreased in 4M6-L and DYNDL13-4-L but showed no significance in 4M6 and DYNDL13-4 compared to the control (Figure 3b). In addition, DYNDL13-4 and DYNDL13-4-L, especially DYNDL13-4-L, resulted in the decrease of other indole derivatives (including 4-indolecarbaldehyde and indole-3-acetaldoxime), kynurenine catabolic metabolites (including xanthurenic acid and 4-(2-aminophenyl)-2,4-dioxobutanoate), and tryptophan derivatives (including 5-hydroxy-dl-tryptophan and *N*-acetyl-dl-tryptophan), most of which were decreased in groups of fecal samples containing lactose consumption, especially DYNDL13-4-L and lactose group. These results indicated that *S. thermophilus* strains differentially altered the catabolic flux of tryptophan in serum. The change was stimulated with continued lactose supplementation, as evidenced by the decreased kynurenine branch and indole derivatives and the increased serotonin. Likewise, serum metabolites of fatty acids were differentially increased in *S. thermophilus* strains consumption, and the lactose supplementation further enriched more fatty acids, such as lignoceric, docosanoic, and palmitoleic acid. These changes were the most prominent in DYNDL13-4-L, and the fatty acids profiles in 4M6-L tended to be those in DYNDL13-4. This suggested that lactose intake may reduce differences in lipid metabolism induced by strain variability.

## 4. Discussion

Most studies just focused on evaluating the protective effect of prebiotic supplementation on the individual strains in the gut microbiota [20,21,22]. In this research, we considered the strain-specific responses to lactose supplements, and the regulation of DYNDL13-4 on the gut microbiota was more affected by lactose than 4M6. Various prebiotics may have a protective effect on the probiotics by enhancing their antioxidant capacity, adhesive capacity, resistance to digestive fluids, anti-bacterial effects, and cross-feeding behavior in the gastrointestinal tract [23,24,25,26]. Thus, it is a reasonable hypothesis that these factors may contribute to the differential regulation of strains by lactose. *Bifidobacterium* was the only one increased genera shared by five treatment groups in our results. This is in accordance with the previous studies that *S. thermophilus* ATCC 19,258 enhanced *Bifidobacterium* in the gut of murine models of intestinal tumorigenesis [1], and lactose could regulate the *Bifidobacterium* in healthy adults independently of donors [27].

Alteration in the gut microbiota composition can change metabolic profiles, affecting host health [28]. We observed that tryptophan metabolism in feces and serum was regulated by four treatment groups (DYNDL13-4, 4M6, DYNDL13-4-L, 4M6-L), and the combination of lactose and strains enhanced more changes, especially in indole derivatives. Evidence from animal studies has suggested that tryptophan metabolism may affect host metabolic health via a host–microbiota interaction [29]. As the tryptophan catabolites by gut microbiota, indole derivatives have been shown to have beneficial effects on host diseases, such as inflammatory bowel disease (IBD) and neurological diseases [30]. It was found that IBD patients have reduced fecal concentrations of the indole-3-acetate [31]. 3-Indolepropionicacid was reduced in the serum of patients with colitis when compared to healthy subjects [32], and oral treatment of indole and 3-indolepropionicacid was found to alleviate colonic inflammation in mice [32,33]. Plasma levels of 3-indole propionic acid were significantly diminished significantly in subjects with Huntington’s disease compared to healthy controls [34]. Additionally, a previous study demonstrated a positive correlation between elevated milk consumption and increased levels of 3-indole propionic acid in the serum of genetically lactase non-persistent individuals. Our results emphasized the regulation of indole derivatives by lactose and DYNDL13-4-L and suggested that dietary milk consumption may affect intestinal and neurological function by modulating tryptophan and its derivatives in the host.

The combination of lactose and *S. thermophilus* strain significantly increased the metabolites of arginine and histidine metabolism than the individual strain in feces in a strain-specific pattern. Arginine has been found to improve intestinal mucosal barrier function and is an essential amino acid for the growth and development of intestinal stem cells [35]. In contrast, higher arginine levels may be associated with an increased risk of ischemic heart disease [36]. Multi-cohort analysis showed that abnormal histidine metabolism was related to the occurrence of colorectal cancer and diabetes [37,38]. Thus, the influence of *S. thermophilus* and lactose, key elements associated with fermented milk, on hosts needs to be verified further, including with altered health models, which would provide insights to facilitate designing personalized fermented milk products as a promising dietary therapeutic strategy for improving host health.

In addition, we observed the most abundant metabolites in feces but no significant alteration in serum in the lactose treatment. On the one hand, physiological changes, including chemical and nutritional gradients, are changed in various regions of the small intestine and colon [39]. Fecal samples are only a snapshot of the gut microbiota and are not comprehensive to represent the circulating chemicals. On the other hand, a serum metabolism analysis showed that the amount of lactose peaked in the blood of the healthy volunteers having consumed milk in the first 6 h and dropped off afterward [40]. Our test for serum metabolism was at least 12 h, and differential metabolites may not be observed in the lactose group. We noted that lactose supplementation remarkably enriched amino acid metabolism in serum compared to the individual strain treatments, such as histidine, arginine, phenylalanine, tyrosine, and tryptophan metabolism. Previous studies have shown that the consumption of fermented dairy products could regulate amino acid metabolism in the serum of humans. Tyrosine, valine, and proline increased in the serum of healthy humans after cheese intake [40]. A higher abundance of plasma proline and phenylalanine were observed after the yogurt intake compared to the milk intake [41]. These studies mostly attributed to the free amino acids released during the fermentation of dairy products. Our research further revealed several amino acid changes in serum may be caused by a combination of lactose and *S. thermophilus* strains.

## 5. Conclusions

Our results showed that the effect of lactose supplementation on the regulation of *S. thermophilus* on the gut microbiota and host metabolism is strain-dependent. Although consumption of the two *S. thermophilus* strains caused similar changes in the fecal microbiota and metabolism, lactose had a greater impact on DYNDL13-4 regulation than it did on 4M6. More specifically, the combination of lactose and 4M6 mainly enriched pathways of d-glutamine and d-glutamate metabolism, alanine, aspartate and glutamate metabolism, and tryptophan and phenylalanine metabolism in feces, whereas 4M6 only enriched tryptophan and phenylalanine metabolism. DYNDL13-4-L had significant effects on sulfur, taurine, and hypotaurine metabolism in feces and on phenylalanine, tyrosine, tryptophan biosynthesis, and linoleic acid metabolism in serum relative to the DYNDL13-4. Our study provided insight into the complexity of how fermented milk product consumption may influence host health through modifications of the metabolites and gut microbiota.

## Data Availability

Data supporting reported results are available upon reasonable request and in accordance with ethical principles.

## Supplementary Materials

The following supporting information can be downloaded at: https://www.mdpi.com/article/10.3390/nu15224767/s1. Figure S1: Effect of lactose supplementation on metabolic profile induced by *S. thermophilus* related to Figure 2 and Figure 3. (a) PLS-DA score plots based on the fecal metabolomic profiles in positive ionization modes. (b) PLS-DA score plots based on the serum metabolomic profiles in negative ionization modes. (c,d) Heatmap of the differential metabolites of each treatment relative to the control in feces (c) and serum (d). Figure S2: (a,b) Venn diagram of all identified metabolites of each treatment relative to the control in feces (a) and serum (b).
