# Supplementary material for: Influence of Lactose Supplementation on Regulation of Streptococcus thermophilus on Gut Microbiota
Source: Nutrients. 2023 Nov 13;15(22):4767. doi: 10.3390/nu15224767 (PMC10675825; doi:10.3390/nu15224767)
Supplement: Supplementary file 1 [file nutrients-15-04767-s001.zip › nutrients-2682048-supplementary.pdf]

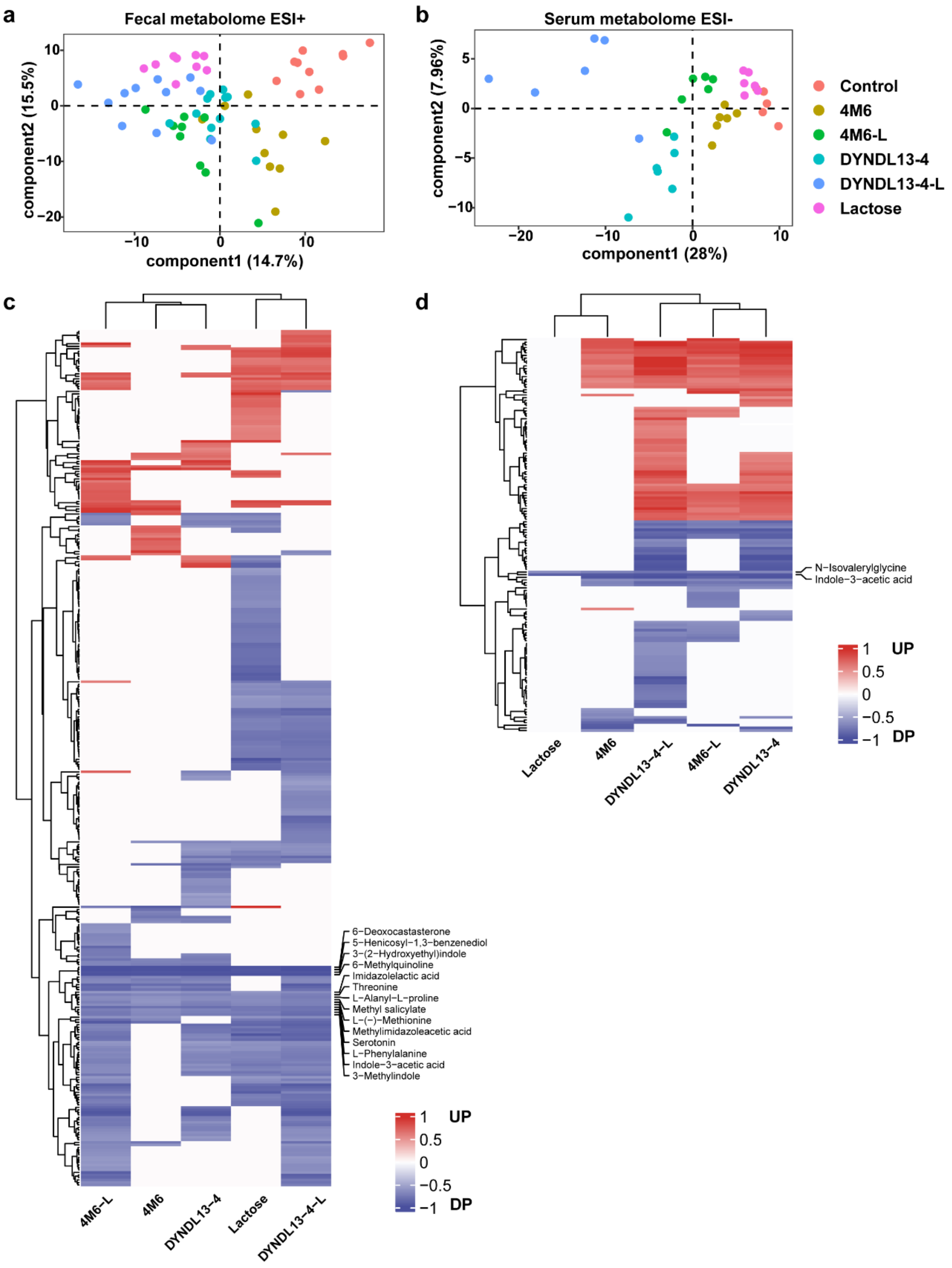

Figure S1. Effect of lactose supplementation on metabolic profile induced by *S. thermophilus* related to Figure. 2 and Figure.3. a, PLS-DA score plots based on the fecal metabolomic profiles in positive ionization modes. b, PLS-DA score plots based on the serum metabolomic profiles in negative ionization modes. c-d, Heatmap of the differential metabolites of each treatment relative to the control in feces (c) and serum (d).

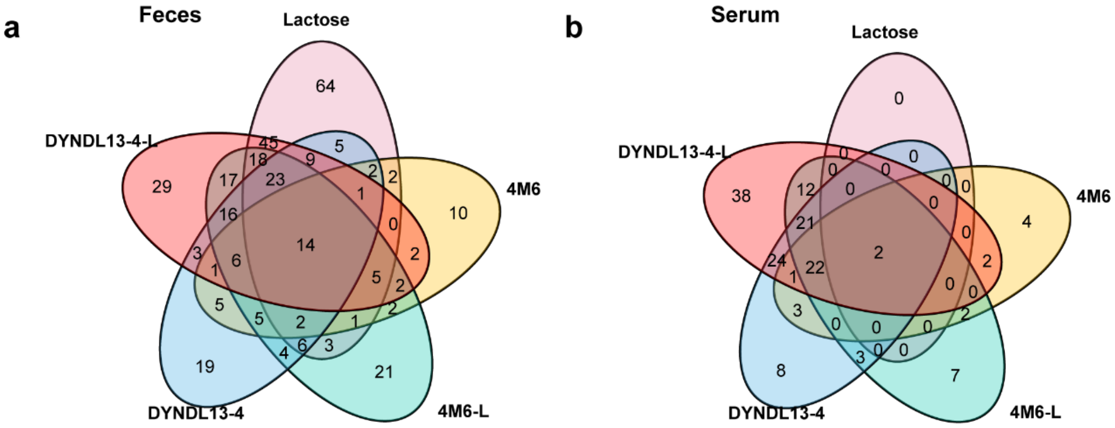

Figure S2. Venn diagram of all identified metabolites of each treatment relative to the control in feces (a) and serum (b).
